# CRISPR/Cas9 Systems for the Development of *Saccharomyces cerevisiae* Cell Factories

**DOI:** 10.3389/fbioe.2020.594347

**Published:** 2020-11-19

**Authors:** Jie Meng, Yue Qiu, Shuobo Shi

**Affiliations:** Beijing Advanced Innovation Center for Soft Matter Science and Engineering, College of Life Science and Technology, Beijing University of Chemical Technology, Beijing, China

**Keywords:** CRISPR/Cas, *Saccharomyces cerevisiae*, cell factory, genetic manipulation, synthetic biology, complex engineering

## Abstract

Synthetic yeast cell factories provide a remarkable solution for the sustainable supply of a range of products, ranging from large-scale industrial chemicals to high-value pharmaceutical compounds. Synthetic biology is a field in which metabolic pathways are intensively studied and engineered. The clustered, regularly interspaced, short, palindromic repeat-associated (CRISPR)/CRISPR-associated protein 9 (Cas9) technology has emerged as the state-of-the-art gene editing technique for synthetic biology. Recently, the use of different CRISPR/Cas9 systems has been extended to the field of yeast engineering for single-nucleotide resolution editing, multiple-gene editing, transcriptional regulation, and genome-scale modifications. Such advancing systems have led to accelerated microbial engineering involving less labor and time and also enhanced the understanding of cellular genetics and physiology. This review provides a brief overview of the latest research progress and the use of CRISPR/Cas9 systems in genetic manipulation, with a focus on the applications of *Saccharomyces cerevisiae* cell factory engineering.

## Introduction

The development of microbial cell factories have drawn increasing attention because they allow the production in a cost-effective, renewable, and sustainable manner (Xu et al., 2020). Ever-expanding genetic toolkits and fundamental understanding have enabled biotechnologists to build or rebuild genetic pathways in many hosts, especially those of model organisms such as *Saccharomyces cerevisiae* (Chen et al., 2017). In the last decades, *S. cerevisiae* has been considered a powerful eukaryotic cell factory for the biosynthesis of many compounds (Brown et al., 2015; Billingsley et al., 2016) or biofuels (Shi et al., 2016b; Yu et al., 2017).

In practice, the Design–Build–Test–Learn (DBTL) cycle has greatly facilitated the construction of an advanced cell factory through designing a genetic modification scheme, building the designated genotypes, testing a rebuilt biosystem at various levels, and learning from systematic data analysis (Liu et al., 2015; Chen et al., 2017). The construction of a successful cell factory always needs several rounds of DBTL cycles due to the complexity of cell metabolism (Billingsley et al., 2016). “Build” can be seen as a key rate-limiting step in the execution of rapid iterative DBTL cycles in generating designated genotypes using traditional genetic tools (Chao et al., 2017). For example, it took more than 250 human years to get a commercial strain for producing farnesene (Karim et al., 2017).

Fortunately, the clustered, regularly interspaced, short, palindromic repeat-associated (CRISPR) system has become an important tool in almost all aspects of synthetic biology and metabolic engineering, including genomic editing, heterologous expression, transcriptional regulation, and genome-wide screening. CRISPR/Cas9 has become the most popular approach in recent years. In CRISPR/Cas9 system, the effector (Cas9) is activated and targeted to specific genomic loci by forming a complex with CRISPR RNA (crRNA) and trans-activating crRNA (tracrRNA) or a single guide RNA that merged from the crRNA and tracrRNA (Jinek et al., 2012; DiCarlo et al., 2013; Mans et al., 2015). Moreover, the development of Cas9 protein variants and the availability of mutually orthogonal Cas9 proteins have greatly maximized its functions and applications (Lian et al., 2017, 2018a; Si et al., 2017). In a word, remarkable improvements in the effectiveness and scope of CRISPR/Cas9 system have made it powerful and versatile for almost all possible genetic manipulations needed for constructing microbial cell factories. Due to its countless applications, two scientists who pioneered the CRISPR technology won the Nobel Prize in Chemistry in 2020^1^.

This review mainly focused on the latest advances of the CRISPR/Cas9 system in the model yeast *S. cerevisiae*. Special attention was paid to examples in four application areas: flexible and precise genetic manipulation, multiplexed editing, transcriptional regulation, and genome-scale engineering/screening. Finally, perspectives on the challenges and opportunities were discussed and highlighted.

## Flexible and Precise Genetic Manipulation

**FIGURE 1:**
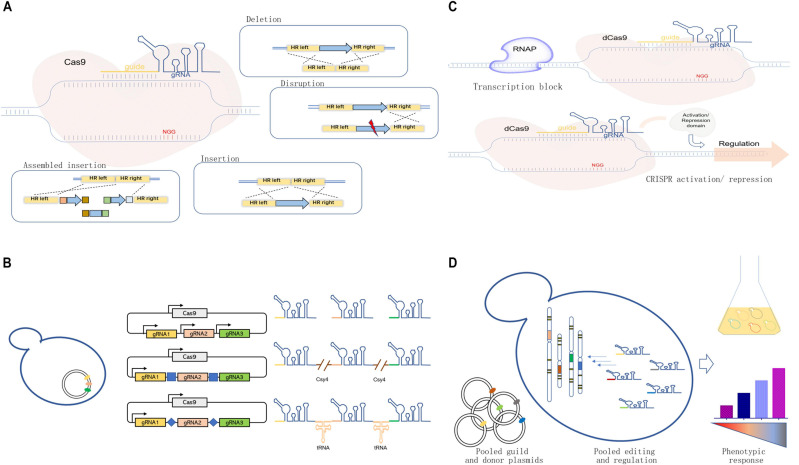
Development and applications of the clustered, regularly interspaced, short, palindromic repeat-associated (CRISPR)/CRISPR-associated protein 9 (Cas9) systems in yeast cell factory engineering. **(A)** Scheme of the CRISPR/Cas9 system for genome editing, including gene deletion, mutation or disruption, insertion, and assembled insertion. **(B)** Guide RNA (gRNA) multiplexing strategies, including multi-single guide RNA (multi-sgRNA) expression through the use of multiple promoters or the expression of one-promoter-guided single transcript separated by different features for RNA cleavage. **(C)** dCas-meditated CRISPRi and CRISPRa systems for transcriptional regulation achieved through physically blocking RNA polymerase (RNAP) or recruiting protein effectors for target repression/activation. **(D)** Pooled gRNA-guided genome-scale engineering or screening.

One of the most significant advantages of the CRISPR/Cas9 system is its flexibility and efficiency for operation with high accuracy. Cas9 protein is a “scissor” to introduce double-strand breaks (DSBs), and guide RNA (gRNA) can be regarded as a “guide” for target-specific recognition (Jinek et al., 2012). The formed DSBs required intrinsic DNA repair mechanisms for editing target loci (Lian et al., 2018a). The homology-dependent recombination (HDR) in yeast can repair DSBs with flexible donors containing desired sequences (Figure 1A), which allows various genetic manipulations, including gene deletion (e.g., whole coding sequence knockout) (Zhang et al., 2019), gene mutation or disruption (DiCarlo et al., 2013), and gene integration (Shi et al., 2016a; Roy et al., 2018).

In the aforementioned processes, strain engineering displayed a high editing efficiency (Table 1). For example, DiCarlo et al. (2013) first demonstrated in yeast that both gene disruption and insertion could be achieved with nearly 100% efficiency using a 90-bp dsOligo as the donor and the CRISPR/Cas9 system. Notably, DNA integration efficiency declined rapidly when the size of the target DNA increased, which could be considered as the limiting factor in integrating large DNA fragments. Using the CRISPR/Cas9, Shi et al. (2016a) developed a Di-CRISPR platform that realized the integration of a 24-kb pathway for the production of (*R*,*R*)-2,3-butanediol. This was a significant achievement in the efficiency and multicopy integration of large DNA.

**TABLE 1:** Selected clustered, regularly interspaced, short, palindromic repeat-associated (CRISPR)/CRISPR-associated protein 9 (Cas9)-associated applications in cell factory construction.

**Types**	**Methods**	**Key features and achievements**	**References**
Flexible and precise genetic manipulation	CRISPR/Cas9 for genome engineering (using 90-bp dsOligo donor)	First achieved site-specific mutagenesis and allelic replacement with nearly 100% efficiency	DiCarlo et al., 2013
	Di-CRISPR (delta integration CRISPR-Cas)	Assembled an unprecedented 18-copy, 24-kb pathway for the production of (*R*,*R*)-2,3-butanediol	Shi et al., 2016a
	CasPER (Cas9-mediated protein evolution reaction)	Employed error-prone PCR and CRISPR/Cas9 system for the directed evolution of key enzymes, resulting in 11-fold higher production of isoprenoids	Jakociunas et al., 2018
	Seamless site-directed mutagenesis	Introduced point mutations at 17 positions by a two-step method and constructed a target mutant for a measurable phenotype	Biot-Pelletier and Martin, 2016
	CHAnGE (CRISPR–Cas9- and homology-directed repair-assisted genome-scale engineering)	Validated single-nucleotide resolution genome editing by creating a genome-wide gene disruption collection with improved tolerance to growth inhibitors	Bao et al., 2018
	Base editor for single-nucleotide replacement using nCas9	Connected cytidine deaminase domain and the nCas9 domain and elicited C-to-T mutations with high accuracy and efficiency	Tan et al., 2019
Multisite editing	HI-CRISPR (homology-integrated CRISPR)	First example of CRISPR/Cas9 multiple disruption in *S. cerevisiae* with efficiency ranging from 27 to 87%	Bao et al., 2015
	CRISPR/Cas9 multiplex genomic editing	Realized quintuple disruption using individual gRNA cassettes in the mevalonate pathway with titers increased more than 41-fold	Jakociunas et al., 2015a
	CasEMBLR (Cas9 facilitated multiloci DNA integration assembler)	Combined *in vivo* assembly and targeted editing, allowing marker-free integration of 15 DNA parts for carotenoid production in 3 loci or 10 DNA parts for tyrosine production in 2 loci	Jakociunas et al., 2015b
	Multiplexed CRISPR/Cas9 genome editing and gene regulation	Exploited bacterial endoribonuclease Csy4 to generate multiple gRNAs from a single transcript and performed a quadruple deletion with 96% efficiency or an efficient regulation of three genes	Ferreira et al., 2018
	GTR-CRISPR (gRNA–tRNA array for CRISPR-Cas9)	Utilized endogenous tRNA-Gly processing to generate multiple gRNAs from a single transcript and disrupted eight genes with 87% efficiency in one step	Zhang et al., 2019
	Lightning GTR-CRISPR	Directly transformed the Golden Gate reaction mix into yeast and disrupted six genes in 3 days with 60% efficiency. Two-round application of Lightning GTR-CRISPR could simplify yeast lipid networks, resulting in a 30-fold increase in free fatty acid production in 10 days	Zhang et al., 2019
Transcriptional regulation for orthogonal control	Multiplex CRISPRi-mediated downregulation	CRISPRi method for simultaneously downregulating seven genes for enhancing β-amyrin production	Ni et al., 2019
	CRISPR-associated RNA scaffolds to generate synthetic multigene transcriptional programs	Realized simultaneous activation and repression of different target genes from a five-gene pathway (VioABEDC) for optimizing the production of violacein	Zalatan et al., 2015
	STEPS (systematically test enzyme perturbation sensitivities)	Established a method for fine-tuned, graded expression of pathway enzymes via dCas9 regulation by varying sgRNA target location, and identified rate-limiting steps, resulting in an increased 3-dehydroshikimate and glycerol production at 7.8- and 5.7-fold, respectively	Deaner and Alper, 2017
	SWITCH: a CRISPR-based system for rapid genetic engineering and pathway tuning	Achieved iteratively alternated genetic engineering and pathway control state for implementing and tuning the pathway for naringenin	Vanegas et al., 2017
	CRISPR-AID: an orthogonal trifunctional CRISPR system	Combined transcriptional activation, transcriptional interference, and gene deletion; the method enhanced the production of β-carotene by 3-fold in a single step and achieved a 2.5-fold improvement in endoglucanase activity in a combinatorial manner	Lian et al., 2017
Genome-scale engineering/screening	CRISPR/Cas9-mediated automated platform for multiplex genome-scale engineering	Iteratively integrated mutation library into the repetitive genomic sequences using robotic automation and optimized diverse phenotypes on a genome scale, such as acetic acid tolerance	Si et al., 2017
	Cas9-mediated integration approach for tuning gene expression	Identified targets that improved protein secretion when expressed at different levels, achieving 2.2-fold improvement in amylase production	Wang et al., 2019
	CHAnGE (CRISPR–Cas9- and homology-directed repair-assisted genome-scale engineering method)	Rapidly created genome-wide disruption mutants for the directed evolution of acetic acid tolerance, achieving a 20-fold improvement	Bao et al., 2018
	MAGIC (multifunctional genome-wide CRISPR)	Combined CRISPR-AID and array-synthesized oligo pools to create comprehensive genomic libraries for obtaining furfural tolerance and surface display levels of endoglucanase, thus facilitating complete genotype–phenotype mapping	Lian et al., 2019

The high efficiency and flexibility also allowed the rapid generation of a mutant library (Table 1). Guo et al. (2018) created hundreds of mutated stains for 315 poorly characterized open reading frames (ORFs) using the CRISPR/Cas9 system, of which 68 were found to be vital for growth. Jakociunas et al. (2018) combined error-prone polymerase chain reaction (PCR) and Cas9-mediated genome integration for protein-directed evolution. The large mutagenized DNA fragments generated by error-prone PCR were integrated into the genome for creating millions of mutants without any bias in mutation frequency. Two mutant enzymes were found, resulting in the increased production of isoprenoids close to 11-fold. Because of its simplicity, flexibility, and high efficiency in knock-in, the CRISPR/Cas9 system enabled the rapid economic development of a high-throughput industrial yeast cell factory that usually required a lot of genomic integration manipulations.

The aforementioned cases showed high precision and accuracy in gene editing using the CRISPR/Cas9 system (Table 1). However, it is initially difficult for the CRISPR/Cas9 system to introduce mutations at single-nucleotide resolution due to its off-target effects (O’Geen et al., 2015). Various strategies have been reported to increase the fidelity and specificity, including well-designed gRNAs (Wang and Coleman, 2019), mutants of Cas proteins (Hu et al., 2018), paired nCas or fCas complexes (Shen et al., 2014; Tsai et al., 2014), and deaminase-dependent strategy (Gaudelli et al., 2017; Tan et al., 2019). For example, a two-step strategy using the CRISPR/Cas9 system was demonstrated to seamlessly introduce 17 precise single mutations in *S. cerevisiae* (Biot-Pelletier and Martin, 2016). Recently, a novel single-nucleotide resolution editing tool was reported (named as CHAnGE) by combining HDR and the CRISPR/Cas9 system that enabled the rapid engineering of *S. cerevisiae* for improved tolerance to growth inhibitors (Bao et al., 2018). Meanwhile, Tan et al. (2019) adopted a deaminase-dependent strategy that could selectively edit a single cytidine at a specific position. These high-precision tools guaranteed the introduction of specific point mutations in genome for genetic diversification, which gained special interest in terms of cell factory development using the bottom–up approach.

The CRISPR/Cas9 system has a significant advantage in its ease and wide applicability (Table 1). Its use is simple in designing and expressing gRNAs. In addition, it has been readily implemented in precise genome editing at an unprecedented level. Furthermore, the lethal characteristic of DSBs introduced by Cas9 endonuclease offers convenience for marker-free positive selection, which is especially useful in non-model microorganisms due to the lack of developed selectable markers.

## Multisite Editing to Accelerate the Building Process

The CRISPR/Cas9 system is suitable for simultaneous multigene editing in *S. cerevisiae* because of the high HDR rate (Table 1). The execution of multigene editing requires the expression of multiple gRNAs (Figure 1B), which can be transcribed individually by RNA polymerase promoters (Jakociunas et al., 2015a; Ding et al., 2020) or transcribed in a single long transcript. Then, individual gRNAs can be released through different strategies.

Using the strategy of individual expression, Jakociunas et al. (2015a) constructed a plasmid harboring multicassettes to express different gRNAs with individual promoters. This approach successfully engineered five genes in one step and achieved a 41-fold improvement in the production of mevalonate. Jakociunas et al. (2015b) further extended and updated this method to CasEMBLR by combining *in vivo* assembly and targeted editing; CasEMBLR allowed a marker-free integration of 15 exogenous DNA parts in one step. Similarly, Ronda et al. (2015) developed CrEdit to manipulate three genomic DNAs by generating three gRNAs, respectively, which completed simultaneous triple insertions of a non-native pathway for β-carotene production in *S. cerevisiae* without selection, with up to 84% targeting efficiency.

Using the single-transcript strategy for expressing gRNAs, homology-integrated CRISPR-Cas (HI-CRISPR) was developed for disrupting three genes simultaneously in the artificial hydrocortisone biosynthetic pathway with an efficiency ranging from 27 to 87%. The pre-crRNAs were transcribed by one promoter and then processed into multiple crRNAs by host RNase III and unknown nuclease(s) (Bao et al., 2015). Ferreira et al. (2018) adopted bacterial endoribonuclease Csy4 for expressing a single transcript containing multiple gRNAs fused with Csy4-cleavable RNA, contributing to a quadruple deletion with 96% efficiency.

Recently, Zhang et al. (2019) developed a gRNA–tRNA array for CRISPR-Cas9 (GTR-CRISPR) using endogenous tRNA^*Gly*^ for gRNA processing; this method disrupted eight genes with 87% efficiency in one step, which is the best example of multigene editing. As a case study, GTR-CRISPR was adopted to obtain a 30-fold increase in free fatty acid production within 10 days.

The aforementioned studies demonstrated the ability to edit multiple genes simultaneously, with varied efficiency. The selection of gRNA sequences and the efficient expression of gRNAs seem to be critical to achieve a high efficiency. It is therefore believed that the multiple-gene manipulation of CRISPR-mediated methods and applications may greatly benefit from the study of gRNA design and efficient expression. The implementation of multiloci editing using CRISPR systems has greatly reduced the timeline of operation. For example, the traditional method requires approximately 6 weeks for editing three genomic loci (Horwitz et al., 2015), while using multiplexed CRISPR-Cas9 needs only 1 week with one transformation step. Moreover, the GTR-CRISPR even achieved six-gene disruptions in 3 days by avoiding the cloning step in *Escherichia coli* (Zhang et al., 2019).

## Transcriptional Regulation for Orthogonal Control

Besides the precise manipulation of genomic DNA, the CRISPR/Cas9 system serves as a transcriptional regulation platform with the adoption of inactive Cas protein (e.g., dCas9, with H840A and D10A mutations, loses its endonuclease activity but retains its capability of sequence-specific binding). Further, dCas9 can be combined with effector domains as artificial scaffolds, thus influencing genomic structure and transcriptional regulation (Lian et al., 2018a; Ding et al., 2020).

As shown in Figure 1C, the CRISPR interference (CRISPRi) used dCas9-mediated DNA recognition complex as a block in physical space to specifically interfere with transcription initiation and elongation (Qi et al., 2013). Based on this strategy, Ni et al. (2019) used a CRISPRi method for downregulating the expression of seven genes simultaneously for enhancing β-amyrin production in *S. cerevisiae*. However, the physical block alone could not always result in an efficient repression. The dCas9 can be fused with several transcriptional repressor domains or chromatin modifiers for effective repression (Figure 1C). In *S. cerevisiae*, the addition of dCas9 fusion domain, Mxi1, could lead to a 53-fold repression compared with 18-fold repression using dCas9 alone (Gilbert et al., 2013). Later, Lian et al. (2017) compared different repression domains in yeast and found that several native repression domains, RD2, RD5, and RD11, worked the best for CRISPRi. Similarly, CRISPR could mediate the transcription activation of target genes (CRISPRa) by recruiting transcription activators with dCas9 (Figure 1C). In *S. cerevisiae*, the recruitment of herpes simplex viral protein 16 (VP64) could lead to up to 70-fold activation by increasing the number of targeting sites (Farzadfard et al., 2013). Later, Chavez et al. (2015) rationally designed a tripartite activator, VP64-p65-Rta, which showed an efficient activating effect in *S. cerevisiae* (∼10-fold). Moreover, modular scaffold RNAs could also be used for CRISPRi and CRISPRa to replace the aforementioned effector domains. For example, Zalatan et al. (2015) developed a modular RNA-based system that enabled the recruitment of activators or repressors by converting the gRNA into a scaffold RNA (scRNA) for transcriptional programming. In addition, the multivalent recruitment with two RNA hairpins could produce a stronger activation effect. It is now feasible to permit programmable transcriptional regulation orthogonally by taking advantage of the binding activity of dCas and different effector domains. Besides, Wang et al. (2019) used the Cas9-mediated integration approach for tuning the transcriptional levels of multiple genes in a combinatorial manner by integrating overexpression cassettes and/or RNAi cassettes without the involvement of effector domains. The developed method was used to optimize the production of amylase.

Functional CRISPR regulatory systems have been exploited simultaneously for combinatorial genetic manipulations (Table 1). One particularly interesting application could tune the expression levels of a five-gene pathway (*VioABEDC*) for optimizing the production of violacein with simultaneous activation and repression (Zalatan et al., 2015). Later, Deaner and Alper (2017) established a new system called systematically test enzyme perturbation sensitivities (STEPS) to achieve a graded expression of target genes by varying gRNA-binding sites in promoter regions. STEPS was used to identify the rate-limiting steps and alleviate pathway bottlenecks, resulting in a 7.8- and 5.7-fold increased 3-dehydroshikimate and glycerol production, respectively. Similarly, SWITCH system was developed to achieve gene integration and regulation simultaneously; it was used to establish and optimize a cell factory for naringenin production (Vanegas et al., 2017). Recently, Lian et al. (2017) established orthogonal trifunctional CRISPR system (CRISPR-AID) that simultaneously enabled gene editing and transcriptional regulation. As proof of concept, this strategy was successfully used to enhance the production of β-carotene by 3-fold and give a 2.5-fold improvement in endoglucanase activity. Combinatorial transcriptional regulation is central to developing yeast cell factories or understanding the complex behavior of synthetic biological systems. It requires not only gain- and loss-of-function genome engineering but also a fine-tuned and programmable control of the expression of multiple genes, so as to engineer or study synthetic biosystems.

## Genome-Scale Engineering/Screening

Libraries of strains with versatile genetic alterations at the genome level could provide invaluable knowledge for understanding genome functions or permitting a direct screening of desired traits. It is still tedious to introduce genome-wide perturbations using available techniques (Lian et al., 2019). Fortunately, the fast development and effectiveness of CRISPR tools permit researchers to build activated and/or interfered gene libraries for genome-wide perturbations in a more standardized and advanced manner compared with previous methods (Table 1). Recently, Si et al. (2017) reported a robotic platform for automated multiplex genome-scale engineering using a standardized workflow. With the aid of CRISPR/Cas9, this platform iteratively integrated standardized genetic parts into repetitive genomic sequences of *S. cerevisiae* and permitted functional mapping and optimization for diverse phenotypes. Wang et al. (2019) incorporated Cas9-facilitated workflow to generate a library comprising RNAi/overexpression (OE) targets for the identification and combinatorial manipulation of the expression levels of favorable gene targets.

It is now possible and convenient to generate a strain library with genetic changes across the whole genome using pooled gRNAs through efficient chip-based synthesis of oligo pools (Figure 1D). Bao et al. (2018) developed a CHAnGE system that could rapidly construct numerous specific genetic variants in yeast. A genome-wide gene disruption was created by this method with an average frequency of 82% and then applied to improve cell tolerance to furfural. Similarly, a gene activation library was created to screen genes for better thermotolerance in *S. cerevisiae*, which identified a key factor in thermotolerance that benefited from *OLE1* (Li et al., 2019). The genome-scale library of gRNAs could also be combined with CRISPRi and CRISPRa to generate genome-wide libraries for silencing or/and activating genes. For example, Smith et al. (2016) combined gRNA libraries with CRISPRi, establishing a screening method for functional and/or chemical genomic screens. Recently, Lian et al. (2019) combined previously reported CRISPR-AID and array-synthesized oligo pools, thus creating a comprehensive and diversified genomic library for gain/reduction/loss of function. The developed system, called multifunctional genome-wide CRISPR (MAGIC), covered almost all ORFs and RNA genes (>99%). It served as a powerful tool to uncover previously uncharacterized gene interactions or engineer complex phenotypes for different biotechnological applications.

The genome-wide CRISPR screening tactics give a significant push to complete genotype–phenotype mapping, analyze complex biological systems, and finally take a big step forward in the metabolic engineering of yeast cell factories. It is important that new knowledge and guidance be gained from the simultaneous activation and repression of various target genes.

## Conclusion and Future Perspectives

CRISPR/Cas9-based tools are considered revolutionary and versatile platforms for genetic manipulations and synthetic biology. This review summarized recent developments and applications of the CRISPR/Cas9 system in the construction and optimization of *S. cerevisiae* cell factory. However, these tools still have some limitations and challenges.

The design and expression of gRNAs is a crucial factor severely affecting editing efficiency between genes. One possible reason could be the formation of secondary structures of gRNAs (Thyme et al., 2016). Usually, several gRNAs should be tested for a new target; however, verifying the target efficiency of each gRNA is a time-consuming process. The predictable accuracy needs further improvement. Some software, websites, rules, and algorithms have been established, for example, Zhang Lab Guide Design Resources^2^, CRISPR direct^3^ (Naito et al., 2015), CHOPCHOP (Montague et al., 2014), and yeast proprietary gRNA tool^4^.

Another key problem limiting further applications of the CRISPR system is the yeast transformation efficiency, especially for multisite integration and genome-scale engineering. A large size and an increased number of adopted donor DNAs might reduce the likelihood to simultaneously enter the cells, thus limiting the use of repair templates for gene editing. It was also revealed that the integration efficiency facilitated by CRISPR could be enhanced if more donor DNA could enter the cells (Shi et al., 2016a). The reported HI-CRISPR (Bao et al., 2015) and multiplexed accurate genome editing with short, trackable, integrated cellular barcodes (MAGESTIC) (Roy et al., 2018) both linked HDR donors with gRNA cassette in one plasmid, providing a useful strategy to facilitate DNA delivery at high efficiency.

The currently adopted activation domain for CRISPRa could only provide a limited activation compared with inducible promoter with upregulated strength up to 1,000-fold (Lian et al., 2018b). Hence, a more efficient activation domain should be screened or engineered, or a novel strategy should be developed to activate genes.

Despite the limitations, the development of the CRISPR system has undoubtedly created a new era for genomic manipulation. The building step is time consuming in the DBTL cycle of cell factory engineering, but CRISPR technology has accelerated this process. Eight genomic edits can be achieved in a week using the CRISPR/Cas9 system, which took several weeks to complete in the past. The CRISPR system might prove to be a more powerful tool in the future when integrated with new design principles learned from genome-scale metabolic models and efficient handling options from automated robotic systems.

## Author Contributions

JM and SS outlined this manuscript. JM drafted the manuscript. SS revised the manuscript. YQ summarized the literature. All authors contributed to the article and approved the submitted version.

## Conflict of Interest

The authors declare that the research was conducted in the absence of any commercial or financial relationships that could be construed as a potential conflict of interest.

## Footnotes

1 https://www.nobelprize.org/prizes/chemistry/2020/press-release/

2 http://crispr.mit.edu/

3 http://crispr.dbcls.jp/

4 http://yeastriction.tnw.tudelft.nl/
